# Self-assisted wound healing using piezoelectric and triboelectric nanogenerators

**DOI:** 10.1080/14686996.2021.2015249

**Published:** 2022-01-07

**Authors:** Fu-Cheng Kao, Hsin-Hsuan Ho, Ping-Yeh Chiu, Ming-Kai Hsieh, Jen‐Chung Liao, Po-Liang Lai, Yu-Fen Huang, Min-Yan Dong, Tsung-Ting Tsai, Zong-Hong Lin

**Affiliations:** a Institute of Biomedical Engineering, National Tsing Hua University, Hsinchu, Taiwan; b Department of Biomedical Engineering and Environmental Sciences, National Tsing Hua University, Hsinchu, Taiwan; c Department of Orthopaedic Surgery, Spine Section, Chang Gung Memorial Hospital, Taoyuan, Taiwan; d College of Medicine, Chang Gung University, Taoyuan, Taiwan; e Institute of Analytical and Environmental Sciences, National Tsing Hua University, Hsinchu, Taiwan; f Material and Chemical Research Laboratories, Industrial Technology Research Institute, Hsinchu, Taiwan; g Department of Power Mechanical Engineering, National Tsing Hua University, Hsinchu, Taiwan; h Frontier Research Center on Fundamental and Applied Sciences of Matters, National Tsing Hua University, Hsinchu, Taiwan

**Keywords:** Wound healing, nanogenerator, piezoelectric effect, triboelectric effect, self-powered system, 201 Electronics / Semiconductor / TCOs < 200 Applications, 202 Dielectrics / Piezoelectrics / Insulators < 200 Applications, 211 Scaffold / Tissue engineering/Drug delivery < 200 Applications, 212 Surface and interfaces < 200 Applications

## Abstract

The complex process of wound healing depends on the coordinated interaction between various immunological and biological systems, which can be aided by technology. This present review provides a broad overview of the medical applications of piezoelectric and triboelectric nanogenerators, focusing on their role in the development of wound healing technology. Based on the finding that the damaged epithelial layer of the wound generates an endogenous bioelectric field to regulate the wound healing process, development of technological device for providing an exogenous electric field has therefore been paid attention. Authors of this review focus on the design and application of piezoelectric and triboelectric materials to manufacture self-powered nanogenerators, and conclude with an outlook on the current challenges and future potential in meeting medical needs and commercialization.

## Grpahical Abstract

**Figure:**
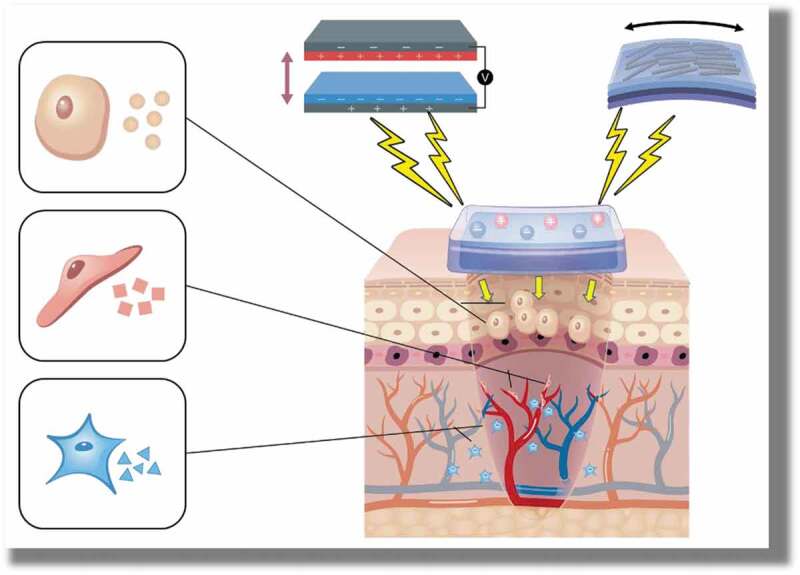

## 1. Introduction

In certain materials, including crystals, ceramics, and biological substances such as bone, DNA, and proteins, electrical charges accumulate owing to applied mechanical stress [1]. Electromechanical interaction is referred to as piezoelectricity and there are two types of piezoelectric effect: direct and inverse. For the direct piezoelectric effect, electrical output is generated by a material under mechanical stress; this output is directly proportional to the applied mechanical stress. The inverse piezoelectric effect is the reverse of this process: a piezoelectric material produces mechanical strain under the application of an electric field (EF).

Piezoelectricity has various industrial applications, and is particularly useful for those involving vibrational generation and actuation. Commercial applications of piezoelectricity include the use of piezoelectric crystals and quartz resonance for time-keeping devices such as microphones, speakers, radio antenna oscillators, hydrophones, and piezoelectric fuel injectors [2]. Lead zirconium titanate (PZT) is a commonly used piezoelectric material because of its malleable physical properties, high piezoelectric coefficient, and low manufacturing cost [3]. Furthermore, polyvinylidene fluoride (PVDF) is the most common piezoelectric polymer because of its low acoustic impedance, high piezoelectric voltage constant, and copolymers such as poly(vinylidene fluoride-tri-fluoroethylene) (P(VDF-TrFE)), which render this material especially suitable for sonar and biomedical applications [4–6]. Various composite and nanostructured materials have also been developed, which can be made into films, discs, or stacked sheets [2,7–9].

As regards biomedical applications, biological systems have structures that do not support the development of many conventional piezoelectric devices. The size limitations and biocompatibility and flexibility requirements of biological systems have prompted investigation of polymers, composites, nanostructures, and lead-free piezoelectric materials. Additionally, understanding of the piezoelectric structure of the human body and its stimulation is necessary for the development of piezoelectric medical technology and biomedical equipment. In the ionic biological system of the human body, charged ions are transported in the tissue, generating potential differences. Several reviews have addressed subcategories of biological piezoelectric materials, such as piezoelectricity in bone [10] and biopolymers [11]. Notably, Fukada et al. [11–13] conducted many original studies examining piezoelectricity in the body, including its presence in the bone, aorta, tendons, and intestines.

Increased awareness of the importance of bioelectricity has yielded the development of electrotherapy for wound healing acceleration, tissue regeneration, musculoskeletal condition improvement, and bone fracture recovery [14]. In particular, chronic, non-healing wounds such as diabetic foot ulcers and pathological scars have physical and psychological impacts on patients [15]. Wound contraction is a basic physiological healing mechanism that reduces such damage; however, poor contraction may decelerate wound healing [16]. Thus, the main goal of trauma treatment is to promote rapid restoration of the anatomical continuity of the skin. This is important because the skin fundamentally prevents infection from the external environment and maintains the homomorphism of the internal environment.

Recent rapid development of energy harvesters for efficient wound healing has met the growing need for wearable piezoelectric- and triboelectric-based devices [17]. Among them, triboelectric nanogenerators (TENGs) combine contact charging and electrostatic induction, and function through local conversion of mechanical actions (such as human body actions) to generate periodic electrical energy. In a previous review, we detailed the mechanism through which bone cells convert external mechanical stimuli into internal bioelectric signals and cause intercellular cytokines from a piezoelectric perspective [18]. In addition, our review introduced piezoelectric and triboelectric materials as self-powered generators, which can promote proliferation and differentiation of osteoblasts through their electromechanical properties. This concept promotes the development of promising applications for tissue engineering and bone regeneration [18]. Many subsequent studies have since contributed to the overall knowledge of the piezoelectric properties of the human body, their origins, and their medical applications.

The present review aims to provide a broad overview of piezoelectric nanogenerators (PENGs) and TENGs and their use in various technologies, especially medical applications for wound healing. The remainder of this review is structured as follows. Section 2 describes the relationship between EFs and the wound healing process, while Sections 3 and 4 discuss piezoelectric materials and their medical applications, respectively. Section 5 introduces nanogenerators and Sections 6 and 7 review PENGs and TENGs for wound healing, respectively. Finally, Section 8 discusses and concludes this review.

## 2. EFs and the wound healing process

The wound healing process involves a series of precisely regulated steps and events that are related to the presence of various cell types in the wound bed [19,20]. This process is characterized by a discrete timeline of physical attributes that constitute the post-trauma repair process. In undamaged skin, the epidermis and dermis form a protective barrier against the external environment. When this barrier is broken, a regulated sequence of biochemical events targeted towards damage repair is activated. Tissue damage itself triggers acute tissue repair, and this process involves four time-dependent phases: coagulation and homeostasis, which begin immediately post injury; inflammation, which is induced shortly thereafter and prepares the wound bed for new tissue growth; the proliferation phase, which initiates a few days post injury and acts to fill and cover the wound; and the remodeling phase, which begins with the formation of scar tissue and can persist for a year or longer [19–21].

**Figure 1.**
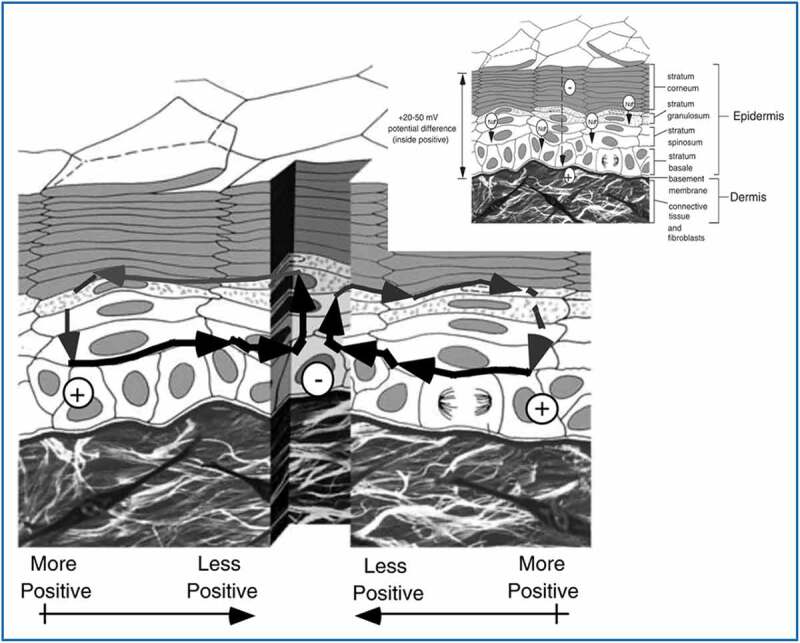
The electric field (EF) generated by the difference in transepithelial potential (TEP) in the damaged epithelial layer regulates the skin-cell behavior and promotes regeneration activity. (Copyright 2003, Elsevier).

Wound management is key to completion of the wound healing process and supporting the skin integrity and function. Regardless of whether the skin wound is acute or chronic, an endogenous EF is generated by differences in the transepithelial potential (TEP) of the damaged epithelial layer. For the wound to heal completely, this state must be maintained until the skin regeneration process is complete [10,22–24]. In detail, the ion gradient at the wound bed generates TEP differences, and TEP destruction yields an endogenous EF [25]. The EF activity then regulates the skin-cell behavior and promotes regeneration activity [22–24,26,27] (Figure 1). The endogenous EF can direct keratinocyte migration [28] and promote angiogenesis [29].

Previous studies have characterized the molecular pathways through which these cells interpret and respond to an EF [30,31]. The therapeutic effect of EF stimulation for wound healing was first observed in the mid- to late 20th century [32,33]. Since then, EFs have been considered essential for direction of cellular processes that naturally promote orderly healing [24,34]. Indeed, electrical stimulation via an electrode can reduce edema around the electrode; stimulate granulation tissue growth; increase blood flow, fibroblast proliferation and collagen production; induce epidermal cell migration; and promote epithelial growth and organization [35]. As regards the development of pre-clinical wound healing applications, EF stimulation has been shown to optimize wound remodeling by promoting more effective fibroblast recruitment and collagen deposition [36–39]. Directed cell migration is essential for wound healing, and EFs arise naturally in biological tissues during development and healing [40]. EFs generated in vitro and in vivo may also guide cell movement through a process called electrotaxis, and in vitro studies have shown that a wide variety of biological cells naturally sense and follow direct-current EFs [41–43]. Some cells migrate toward the cathode (e.g. fibroblasts, keratinocytes, and epithelial cells) [29,30,44,45], whereas others (e.g. endothelial cells) migrate toward the anode [46,47]. These electrical mechanisms contribute to cell localization in various physiological environments [41–43].

Although the strategy of electrical stimulation at a wound site is compelling, clinical application of EF for wound healing involves a large extracorporeal device that induces an EF at the injury site, for which hospitalization may be required. Thus, introduction of wearable and self-powered electrical stimulation devices for wound care in clinical applications would be convenient. If exogenous EF could be provided at a wound site without the use of external electrical equipment, widespread clinical application of EF therapy for wound healing could be realized [48].

## 3. Piezoelectric materials

The various piezoelectric materials are composed of naturally occurring and synthetic materials. Some of the natural materials include crystals (such as quartz, Rochelle salt, topaz, and tourmaline group minerals) as well as organic substances (e.g. silk, wood, enamel, bone, hair, rubber, and dentin) [12,49–52]. Most piezoelectric materials are anisotropic dielectrics with non-centrosymmetric lattices. Synthetic piezoelectric materials are prepared from materials with ferroelectric properties and can be divided into five main categories: quartz analogs [53], ceramics [53–55], polymers [53,56], composites [57,58], and thin films [59,60]. The most common synthetic materials are PZT and PVDF. PZT is a ceramic perovskite material and its family contains the best piezoelectric and ferroelectric properties for integration into devices such as piezoelectric actuators, sensors, and transducers. PVDF is a non-reactive thermoplastic fluoropolymer that is widely used in applications requiring purity [29]. Unlike other piezoelectric materials, PVDF compresses rather than expanding when exposed to an EF because of its negative d33 value [61]. Piezoelectric materials can also be divided into polycrystalline piezoelectric polymers and piezoelectric ceramics, which can be used alone or as composite materials for tissue engineering [19].

## 4. Medical applications of piezoelectric materials

Piezoelectric materials support various electronic applications such as transducers, actuators, and sensors. They also have important applications in tissue engineering as electroactive scaffolds for tissue repair and regeneration, and can provide variable electrical stimulation under mechanical stress with no external power source [62]. The electrical stimulation generated by piezoelectric scaffolds promotes tissue regeneration and repair in a certain manner [63]. In addition, piezoelectric scaffolds with optimized characteristics have been implemented during bone and cartilage remodeling; these scaffolds can generate suitable bioelectric signals, similar to those produced by the natural extracellular matrix [62].

In biomedical applications, the choice of material mainly depends on the strength of the piezoelectric effect and the material cost. The most common piezoelectric ceramics are PZT and quartz. PZT is cheap to manufacture, its piezoelectric coefficient is high, and it has broad applications as its composition can be adjusted. However, quartz is more stable over an extensive temperature range, maintaining consistent characteristics [3]. Restrictions apply as regards development of implants or technology involving direct human contact. Lead-free ceramics, such as quartz, barium titanate, and potassium sodium niobate, have higher biocompatibility [64]. In addition, many biomedical devices require flexibility to accommodate the dynamics of human movement. PVDF and its copolymers are widely used biocompatible polymers with high flexibility, low weight, low thermal conductivity, high chemical corrosion resistance, and heat resistance, and have been developed as biomechanical energy harvesting systems, sensors, and wound scaffolds [64,65].

## 5. Nanogenerators

Nanogenerators have been investigated since the mid-2000s, and comprehensive studies on the harvesting of mechanical energy through application of nanomaterials and nanostructures have been performed. Most nanogenerators are based on one of two physical effects: piezoelectricity, which involves generation of electric dipole moments in a solid crystal structure due to non-centrosymmetry of the crystal structure caused by external forces; and triboelectrification, which occurs when contact between two materials causes charge transfer and triboelectric potential alteration. From these early studies, the new research field of nanoenergy was derived [66,67]. Nanoenergy has been successfully applied in numerous fields to support the self-powered operation of many electronic devices and equipment [67–70]. Following years of research, nanogenerators have been introduced to the field of biomedicine, with applications including healthcare monitoring [71,72], bio-sensing [71], microorganism disinfection, drug delivery [73], and tissue/organ electrical stimulation [74]. The two most broadly used nanogenerators, PENGs and TENGs have attracted particular research focus.

### 5.1. Piezoelectric nanogenerator (PENG)

A PENG is defined as an energy-harvesting device with the ability to convert ambient energy (e.g. mechanical, solar, or thermal power) into electric energy through the action of nanostructured piezoelectric materials. The term ‘PENG’ was first introduced by Wang and Song in their 2006 report on piezoelectricity in zinc oxide (ZnO) nanowire-based nanogenerators [75]. PENG-based devices subsequently attracted considerable research attention in the context of biomedicine and clinical application as self-powered medical bio-devices. PENGs, which can supply real-time electrical stimulation to a wound site in response to an external mechanical force, have been explored in wound management because of their miniaturized size and efficient electricity conversion. Flexible, bendable, non-toxic, biocompatible, and biodegradable piezoelectric materials, including ZnO, barium titanate (BaTiO_3_, BTO), PZT, PVDF, and MoS_2_, have attracted considerable attention for their potential application in biomedical nanogenerators [76,77].

*ZnO-based PENGs*: ZnO has strong piezoelectric properties and is most commonly used in nanogenerators for tissue engineering. The combined semiconducting and piezoelectric characteristics of ZnO nanowires are notable, along with their ability to generate rectifying piezoelectric currents [78]. They can be fabricated on various conducting copolymer coatings [79].

*BTO-based PENGs*: BTO is an inorganic compound with high biocompatibility and cytocompatibility. It is lead-free and is usually embedded into composites to reinforce PENG performance [80].

*PZT-based PENGs*: PZT exhibits marked piezoelectric effects and has numerous clinical applications, such as in ultrasonic transducers and piezoelectric resonators. However, because of concerns regarding lead cytotoxicity, it cannot be used in wound-management and implantable devices.

*PVDF-based PENGs*: PVDF and its copolymers, P(VDF-TrFE), are widely used in the fields of biomedicine, tissue engineering, and implantable self-powered devices because of their high flexibility and lack of cell toxicity. They also demonstrate high chemical and physical resistance [64].

*Two-dimensional PENGs*: The first two-dimensional (2D) PENG with a single atomic layer of MoS_2_ was introduced by Wu et al. in 2014 [81]. Because of the extremely low thickness of the atomic unit, composites using 2D PENGs are suitable for insertion inside the body and applicable to wearable electronic devices.

For flexible PENG devices, the electrodes for nanowire connection and energy output should have high flexibility. Graphene, indium tin oxide (ITO), and metal are candidates for flexible electrode materials.

### 5.2. Triboelectric nanogenerator (TENG)

**Figure 2.**
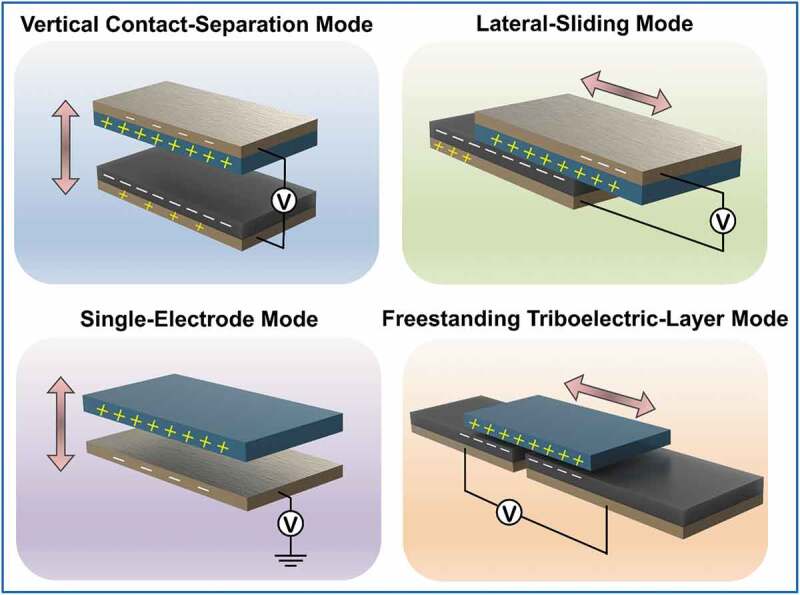
Four triboelectric nanogenerator (TENG) operational modes: the (a) vertical contact-separation (CS), (b) lateral sliding (LS), (c) single-electrode (SE), and (d) freestanding triboelectric-layer (FT) modes. (Copyright 2018, John Wiley and Sons).

The key concept of a TENG is triboelectrification, which is the most common naturally occurring electrical phenomenon. Contact between two dissimilar materials causes electron transfer from one material to the other owing to their different electron attraction capacities. When the surfaces of the two different materials are separated, the contact-induced triboelectric charges create a potential drop. The electric potential then drives the electric currents in the electrodes attached to the materials [18]. Therefore, the mechanical energy used to separate and contact the two dissimilar materials transforms into electric currents. The research group of Dr. Z.L. Wang first demonstrated TENG energy harvesting via contact between a polymethylmethacrylate (PMMA) layer and polyimide nanowires in 2012. Those researchers reported an instant electric power density of 31.2 mW/cm^3^ at a 110-V open circuit voltage [82]. Since then, practical TENG applications have been intensively investigated. TENGs exhibit four operational modes of triboelectrification, i.e. the vertical contact-separation (CS) (Figure 2(a)), lateral sliding (LS) (Figure 2(b)), single-electrode (SE) (Figure 2(c)), and freestanding triboelectric-layer (FT) modes (Figure 2(d)) [83]. Each of these modes is discussed individually below.

*Vertical contact-separation (CS) mode*: The first prepared TENG mode is the vertical CS mode, which is also one of the two basic TENG modes. These TENGs are composed of a pair of triboelectric layers arranged in a face-to-face vertical manner, with electrodes attached to their back sides to form an external circuit (Figure 2(a)). Oppositely charged surfaces are induced in response to the triboelectric effect when two different materials are forced into first contact. The surface charges neutralize each other and the electric potential disappears after the moment of contact. The electrical potential is created again following separation of the two triboelectric layers by a mechanical force. Then, the free electrons in one electrode move to the other electrode to build an opposite potential in order to balance the electrostatic field. When the layers are pushed into contact again, the opposite charges once again neutralize each other and the current flows in the opposite direction, thereby producing an alternating current [84].

*Lateral sliding (LS) mode*: The LS mode is the other of the two basic TENG modes; its mechanism is shown in Figure 2(b). Here, electron movement is triggered by relative sliding between the two triboelectric layers. The resultant EF induces opposite charges on the electrodes to form a current flow in the external circuit. An alternating current is generated in the external circuit by the periodic sliding motion of the triboelectric layers [84].

*Single-electrode (SE) mode*: The SE mode has the same basic mechanism as the CS and LS modes, but with more extensive application. Unlike the CS and LS modes, the SE-mode TENG consists of one triboelectric layer with one attached electrode (Figure 2(c)). This design significantly expands the potential biomedical applications of TENGs, as a moving object can directly serve as the other triboelectric layer [84].

*Freestanding triboelectric-layer (FT) mode*: Figure 2(d) demonstrates the structure of the FT mode. As in the case of the SE mode, the FT-mode TENG was developed to harvest mechanical energy from moving objects. In this TENG, the electrodes are connected to non-moving triboelectric layers, and an asymmetric EF is created on the non-moving triboelectric layers by moving objects. Hence, an electric current is generated between the two attached electrodes to balance the local EF [84].

## 6. PENG application for wound healing

**Figure 3.**
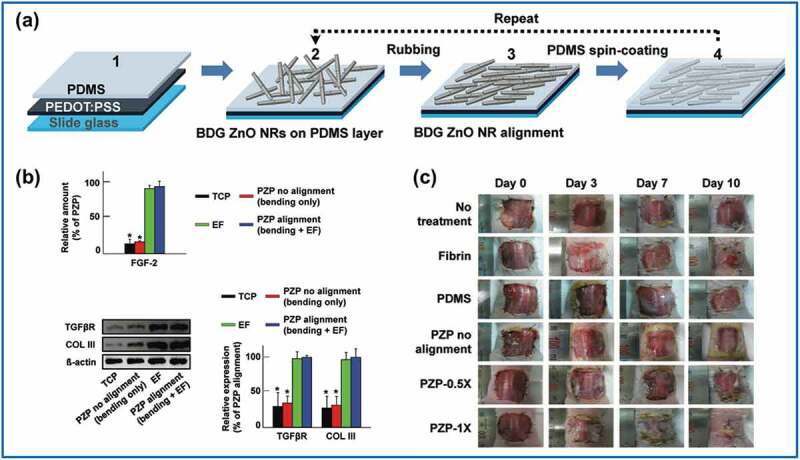
(a) PENG skin patch featuring aligned zinc oxide (ZnO) nanorods on polydimethylsiloxane (PDMS) matrix. (b) Enhanced fibroblast growth factor (EGF-2) production and gene expressions of transforming growth factor-β (TGF-β), TGF-β receptor, and collagen type III. (c) Wound healing promoted by ZnO-based piezoelectric patch. (Copyright 2016, John Wiley and Sons).

Application of self-powered nanogenerators to wound healing involves use of their electromechanical properties to produce real-time electrical stimulation at the wound, thereby enhancing the wound healing process on the cellular level. The value of PENGs for wound management is particularly indicated by their miniaturization and efficient electricity conversion [85–87]. For wound healing, PENG materials must be biocompatible to support continuous cell growth and proliferation [88]. As noted above, ZnO is known to be both piezoelectric and biocompatible. In 2016, a piezoelectric skin patch composed of bidirectionally aligned ZnO nanorods on a polydimethylsiloxane (PDMS) matrix was illustrated by Bhang et al. (Figure 3(a)) [31]. According to that study, electric potentials of 320 and 900 mV were generated with 54.8% and 95.2% ZnO filling density, respectively, through application of ZnO-based piezoelectric patches in animal models. The appropriate electric voltage range for wound healing enhancement is between 150 and 1200 mV [89]. In the in vitro study performed by Bhang et al. [31], the developed ZnO-based piezoelectric patch introduced such an electric potential with only a 20-mm bending radius. The EF generated by bending of the ZnO-based piezoelectric patch (bending radius: 20 mm; frequency: 0.5 Hz) also appeared to promote dermal fibroblast migration and to enhance fibroblast growth factor (EGF-2) production and gene expression (transforming growth factor-β (TGF-β), TGF-β receptor, and collagen type III) in in vitro studies (Figure 3(b)). Significant therapeutic efficacy was also observed for the ZnO-based piezoelectric patch in animal models. In particular, the group with 95.2% ZnO-nanorod filling density exhibited the best wound healing improvement (Figure 3(c)). In a similar study by Augustine et al., a nanocomposite consisting of ZnO and P(VDF-TrFE) exhibited enhanced cell adhesion, proliferation, and angiogenesis, which is promising for tissue engineering and wound healing [90].

**Figure 4.**
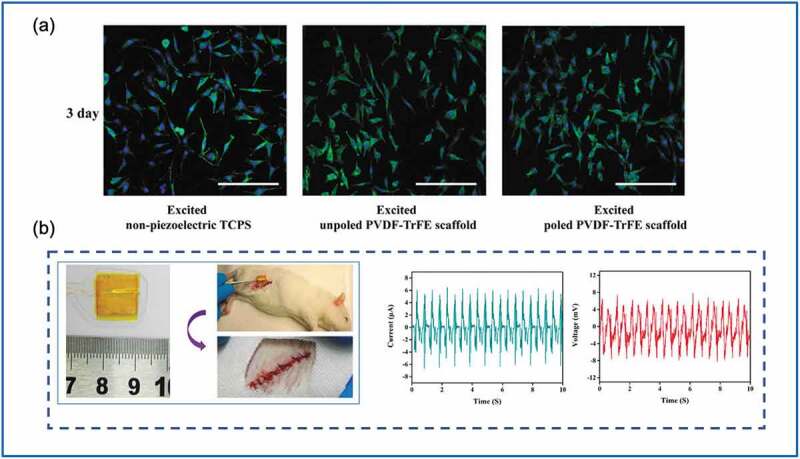
(a) Poly(vinylidene fluoride-tri-fluoroethylene) (P(VDF-TrFE)) nanofiber scaffolds increased fibroblast cell proliferation rate by 1.6 times. (b) In an animal study, the P(VDF-TrFE) nanofiber scaffolds generated a maximum output of 6 mV and ~6 μA through the natural body activity and physiological environment of rats. (Copyright 2018, Elsevier).

A review of the literature reveals that PVDF and its copolymers, P(VDF-TrFE), have been well explored owing to their high piezoelectricity, chemical stability, ease of processing, and biocompatibility [91]. Notably, Guo et al. prepared polyurethane (PU)/PVDF scaffolds through electrospinning, in which the PVDF crystalline phase was changed from the nonpiezoelectric α phase to the piezoelectric β phase. The piezoelectric effects of the scaffolds promoted cell migration, adhesion, and fibroblast secretion, especially under an intermittent 8% deformation at 0.5-Hz frequency. In an in vivo study, PU/PVDF scaffolds implanted into rats accelerated wound fibrosis, which is considered to enhance wound healing [92]. Other piezoelectric scaffolds consisting of P(VDF-TrFE) nanofibers were prepared to modify fibroblast alignment and proliferation in wound healing, as reported by Wang et al. [85]. These P(VDF-TrFE) nanofiber scaffolds increased the fibroblast cell proliferation rate by 1.6 times (Figure 4(a)) with the electric output exceeding 1.5 V (peak-to-peak) and approximately 52.5 nA (peak-to-peak). In an animal study involving rats, the P(VDF-TrFE) nanofiber scaffolds generated a maximum output of 6 mV and ~6 μA from the natural body activity and physiological environment of the rats (Figure 4(b)). The most recent study on PVDF piezoelectricity for wound healing was reported by Du et al. [87], who developed a bioinspired hybrid patch with a PVDF-based PENG to accelerate wound healing. In that work, PVDF nanofibers were aligned on a mussel-inspired hydrogel matrix to construct a bioinspired hybrid patch. An in vitro study revealed that the bioinspired hybrid patch promoted fibroblast proliferation and migration and increased collagen deposition, angiogenesis, and re-epithelialization. The therapeutic efficacy was further demonstrated in an animal study, with an observed wound closure time reduction of approximately one third. Those authors concluded that this PENG-based bioinspired hybrid patch can serve as a wearable and real-time electrical stimulation device, which is potentially useful in clinical applications of skin wound healing.

**Figure 5.**
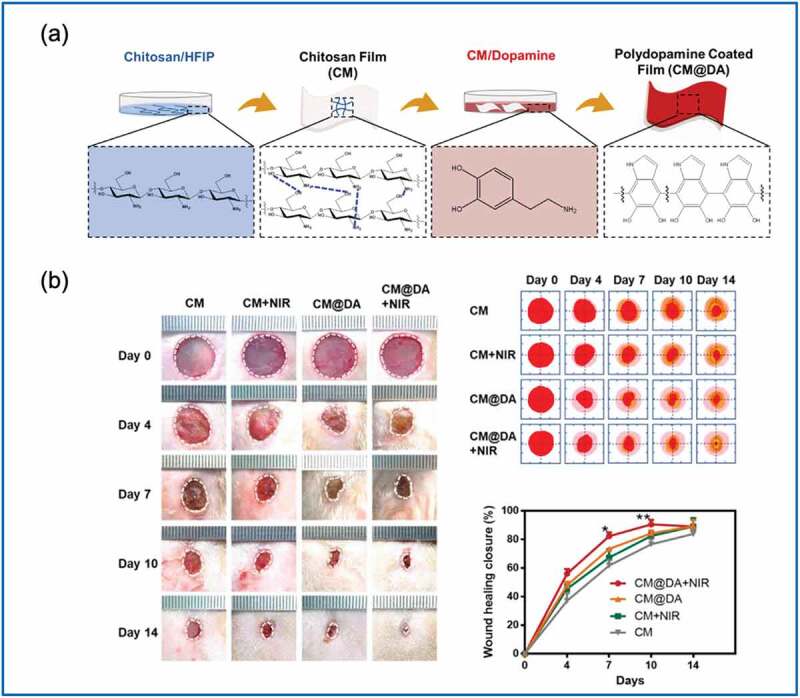
(a) Polydopamine coated on a chitosan film (CM@DA). (b) Increased wound healing rates were observed for rats in the CM@DA plus near-infrared (NIR) irradiation group. (Copyright 2020, Elsevier).

In addition to electric stimulation, thermal therapy is also considered effective for wound healing promotion [93]. Hyperthermia can increase blood flow, enhance enzyme activity, and activate cellular signaling pathways, which eventually stimulate wound healing and tissue repair [94]. Piezoelectric composites for enhanced wound healing, which were fabricated by coating polydopamine on a chitosan film (CM@DA, Figure 5(a)), were prepared by Chen et al. [95]. The CM@DA was triggered to generate an electric potential under kinetic force and exhibited photothermal phenomena under near-infrared (NIR) light irradiation. The CM@DA upregulated expression of heat shock protein 90 and hypoxia-inducible factor 1α, thereby accelerating granulation formation, collagen deposition and maturation, angiogenesis, and re-epithelialization. In rats, the wound healing rate was also increased in the group treated with CM@DA plus NIR (Figure 5(b)). Recently, piezoelectric materials have been used to promote photoinduced electric flow [96,97]. In particular, Yu et al. [97] illustrated use of a piezoelectric material, BaTiO_3_, to enhance reactive oxygen species generation via the photodynamic performance of the TiO_2_/BTO/Au heterostructure. Hence, bacterial killing and wound healing were facilitated. Table 1 summarizes studies on PENGs for wound healing and their key findings.

**Table 1.** Summary of PENGs for wound healing

Piezo materials	Structure	Features	Ref
ZnO NRs*	ZnO NRs aligned on PDMS*	Bidirectionally grown ZnO NR-based piezoelectric patch In vitro: enhanced skin-cell regenerative activity In vivo: enhanced wound healing in rats	[31]
ZnO, P(VDF-TrFE)*	P(VDF-TrFE)/ZnO nanocomposite scaffolds	In vitro: higher cell viability, adhesion, and proliferation In vivo: angiogenesis promotion	[90]
PVDF*	Polyurethane/PVDF scaffolds	In vitro: enhanced fibroblast migration, adhesion, and secretion In vivo: higher fibrosis level	[92]
P(VDF-TrFE) nanofibers	P(VDF-TrFE) nanofiber scaffolds	In vitro: 1.6-fold increase in fibroblast cell proliferation rate; electric output: 1.5 V, 52.5 nA In vivo: maximal 6-mV and ~6-μA electric output via rat leg motion	[85]
PVDF nanofibers	Bioinspired hybrid patch with PVDF nanofibers aligned on mussel-inspired hydrogel matrix	In vitro: fibroblast proliferation and migration promotion, facilitating collagen deposition, angiogenesis, and re-epithelialization In vivo: mouse wound closure time reduction of approximately one third	[87]
Polydopamine	Polydopamine coating on chitosan film (CM@DA)	In vivo: wound regeneration promotion in rats via upregulation of Hsp90 and HIF-1α* expression	[95]
BTO*	TiO_2_/BTO/Au heterostructure	In vitro: photodynamic bacteria killing via ROS* generation In vivo: enhanced infected wound healing	[97]

*NR: nanorod; PDMS: polydimethylsiloxane; P(VDF-TrFE): poly(vinylidene fluoride-tri-fluoroethylene); PVDF: polyvinylidene fluoride; BTO: BaTiO_3_; Hsp90: heat shock protein 90; HIF-1α: hypoxia-inducible factor 1α; ROS: reactive oxygen species

## 7. TENG application for wound healing

As TENGs have the advantage of being self-powered through conversion of mechanical force to electrical energy, their use as efficient energy harvesters has been investigated over the last decade. The mechanical energy output can be obtained from either the outer environment, e.g. from wind, water, raindrops, and vibrations, or the inner environment of the human body, e.g. from movement generated by the diaphragm, heartbeat, and digestion. TENGs that can be worn on the surface of the body have limited applications; however, implantable TENGs have an advantage as regards physiological data sensing and in vivo self-powering. Implantable TENGs are classified as robust or biodegradable devices based on their intended application for permanent or transient use [98]. As electrical stimulation accelerates wound healing, the concept of a wearable/implantable TENG for wound healing seems eminently sensible. Table 2 summarizes the current studies on TENGs for wound healing and their key findings.

**Table 2.** Summary of TENGs for wound healing

TENG design	Features	Materials	Operation mode	Output	Ref
TENG-based bandage	Electric potential generated by rat breathing	Cu/PTFE*	Lateral sliding	∼0.2 V, deep anesthesia; ∼1.3 V, calm; ∼2.2 V, active	[14]
Rotatory disc-shaped TENG	Constant high-voltage output with alternating current via rotational speed	Cu/PTFE	Freestanding triboelectric layer	~160 V/100 μA at 140 rpm	[99]
Wearable ionic TENG patch	Biocompatible, driven by rat skin contact/separation	Stretchable gel-based fabric woven from organogel-filled silicone microtubes	Vertical contact separation	25–75 V at 0.5–2 Hz under pushing tester; ~2 V for active mouse motion	[100]
Surface-engineered TENG patches with drug loading	ES* and controlled drug loading/release for infected wound healing acceleration	PTFE/ Mg-Al LDH@Al*	Vertical contact separation	AC voltage (0.5–4.5 V) and cur- rent (5–40 nA) induced by mouse motion	[101]

* ES: electric stimulation; PTFE: polytetrafluoroethylene; LDH: layered double hydroxide

**Figure 6.**
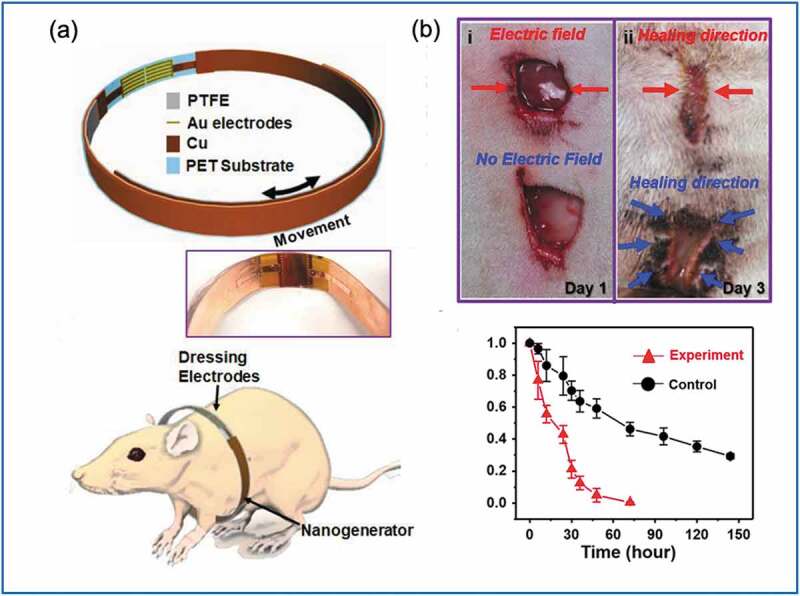
(a) Biomechanical energy conversions of sliding-mode TENG and dressing electrodes of self-activating TENG bandage. (b) The wound closure rate was accelerated in the TENG group. (Copyright 2018, ACS).

In 2018, Long et al. [14] reported a wearable TENG device for wound healing in rats. This device revealed the potential of converting energy harvested from the mechanical breathing pattern to an EF, which was detected through dressing electrodes; hence, the wound healing process was accelerated. This self-activating electrotherapy bandage had two main components: a biomechanical energy converter (a sliding-mode TENG) and dressing electrodes (Figure 6(a)). By overlapping an electronegative Cu/polytetrafluoroethylene (PTFE) layer with an electropositive Cu layer on the opposite side of a polyethylene terephthalate (PET) substrate, the multilayer device (PET-Cu-PTFE) was obtained, which exhibited a lower bending modulus than pure PET material, along with better adaptability to the skin surface. This TENG device converted the mechanical movement of the chest wall during breathing to an approximate electric potential of 0.2–2.2 V. In an in vitro study, migration, proliferation, and differentiation of the fibroblast cells was increased. Furthermore, in vivo wound closure was accelerated to within 3 days for the TENG group compared to the 12 days required by the normal-healing group (Figure 6(b)).

**Figure 7.**
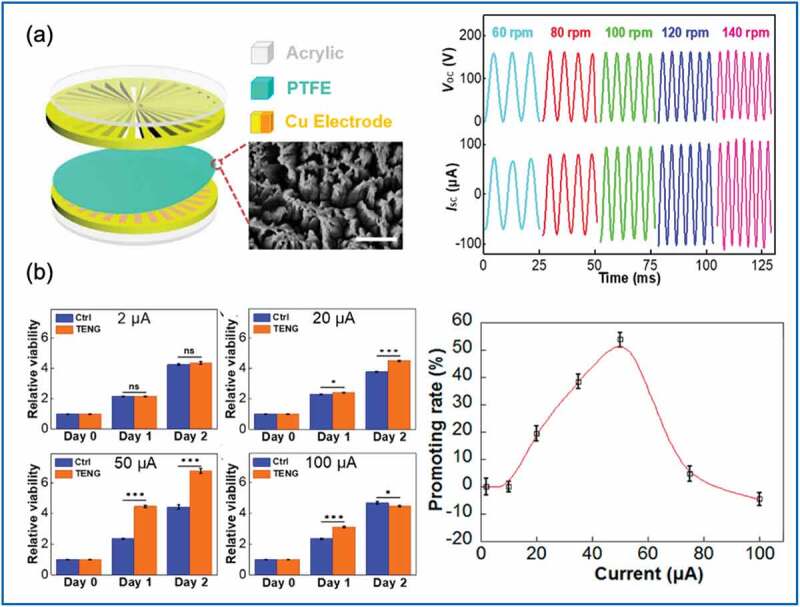
(a) Structure of rotatory disc-shaped TENG (RD-TENG). (b) In vitro, enhanced fibroblast proliferation and migration were observed with RD-TENG current output of 10–50 μA. (Copyright 2019, Elsevier).

In a 2019 study, Hu et al. [99] also targeted fibroblast proliferation and migration behavior under the influence of TENGs. The electric stimulation system employed in that study had three components: a rotatory disc-shaped TENG (RD-TENG), a rheostat, and a cell-culture dish containing mouse fibroblast cells. The RD-TENG consisted of a disc-shaped rotator and a stator, which produced an adjustable range of alternating electric currents at different rotation speeds. The rotator was a radial-Cu-deposited printed circuit board (PCB) and the stator was another PCB-coated interdigital Cu electrode adhered to a PTFE film (Figure 7(a)). The working mechanism of this rotatory RD-TENG was the coupling effect of the triboelectricity and electrostatic induction, with the radially patterned Cu and PTFE film serving as triboelectric layers. The rotatory RD-TENG generated a peak-to-peak open-circuit voltage of approximately 160 V, with a short-circuit current that was adjustable depending on the rotation speed (e.g. 70 and 100 μA at 60 and 140 rpm, respectively). The study results revealed that the developed RD-TENG promoted cell proliferation and migration under a current output of 10 to 50 μA (Figure 7(b)). Under a 50-μA output current, proliferating cell nuclear antigen (Pcna), also known as the initiation gene for cell proliferation, exhibited a significant upregulation of 131%. Migration-related genes, including fibroblast growth factor 2 and delta, like the non-canonical Notch ligand, also exhibited the same tendency toward significant gene up-regulation in the RD-TENG stimulation group.

**Figure 8.**
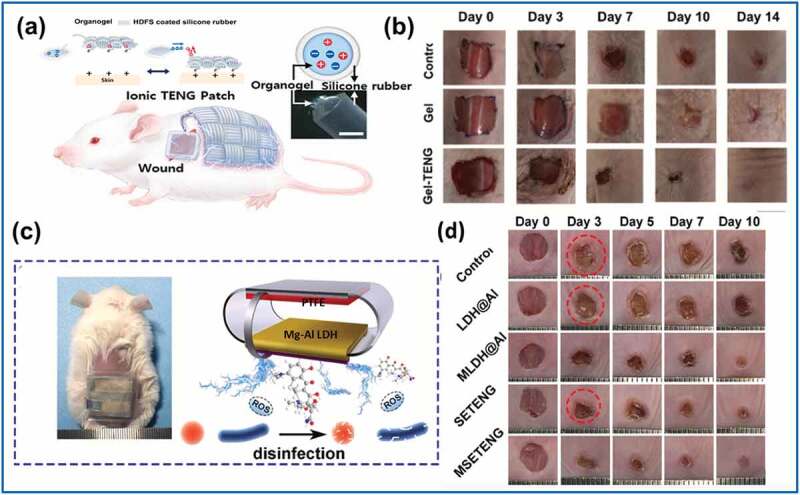
(a) Structure and working mechanism of ionic TENG. (b) Significant wound closure to 40% on Day 3 for Gel-TENG. (c) Design of drug-loaded TENG composed of polytetrafluoroethylene (PTFE) and Mg-Al LDH@Al film (LDH: layered double hydroxide) as the electrode and minocycline container, respectively. d) Rapid infected-wound healing process accentuated by antibacterial and electric stimulation efficacy of drug-loaded TENG (MSETENG). (Copyright 2021, Elsevier).

A wearable textile-type ionic TENG patch for wound healing was introduced by Jeong et al. in 2021 [100]. In that study, organogel-filled silicone microtubes were woven into satin fabrics to act as a stretchable and wearable ionic TENG. Lithium chloride was also incorporated, which increased the ionic conductivity of the organogel. A hydrogel-based patch was connected to the ionic TENG and served as both an electrode and wound dressing. Electric stimulation of the hydrogel-based patch was achieved via an alternating electric current, which was generated through the friction between the skin and ionic TENG (Figure 8(a)). During active movement of mice, discrete voltage spikes were detected through the ionic TENG patch. As regards the in vitro dermal fibroblast cellular response, the ionic TENG yielded significantly enhanced migration ratios of 3.5 and 1.8 times those of the control group for human dermal fibroblasts (HDFs) and diabetic mellitus fibroblasts (DMFs), respectively. Live cell staining, MTS assay, and fluoroscent images all revealed increased cell viability for specimens subjected to 20 h of ionic TENG stimulation, yielded an approximately 2-fold proliferation. Jeong et al. [100] also analyzed the influence of ionic TENGs on wound-healing growth factors using ELISA. Increasing fibroblast growth factor, vascular endothelial growth factor, and epidermal growth factor expression was observed in HDFs and DMFs subjected to ionic TENG electrical stimulation; this outcome indicates the potential application of ionic TENGs to promote angiogenesis and re-epithelialization in both normal and pathologically poor healing wounds. In an in vivo mouse model, the 14-day healing process of a full-thickness wound was tested for three groups: a control group without treatment, a gel group for which the gel was covered with an ionic patch, and a Gel-TENG group in which the ionic patch was combined with TENG electrical stimulation. On Day 3, significant wound closure to 40% of the original size was noted for the Gel-TENG group, whereas the wound closures of the control and Gel groups remained at 80% (Figure 8(b)). Histological examination with hematoxylin and eosin (H&E) and Masson’s trichrome (M-T) staining also indicated an enhanced epithelial layer and regenerated collagen density in the Gel-TENG group compared to the control and Gel groups. Additionally, immunohistochemical analysis revealed a significant increase in alpha-smooth muscle actin expression, which prompted myofibroblasts for wound shrinkage, and CD 34, which acted as an angiogenetic marker, in the Gel-TENG group.

In the case of poor wound healing, wound infection is the major concern. In this context, Du et al. [101] proposed a drug-loaded TENG patch with a surface-engineered electrode possessing Mg–Al layered double hydroxides (LDH) in 2021. The drug-loaded TENG was designed as an arch-shaped patch composed of PTFE (electronegative) and Mg–Al LDH@Al film (electropositive) as the electrode and minocycline container (Figure 8(c)). An alternating current was induced via contact and separation between the two fabric materials by an external force. Electrical stimulation was found to help wound healing and promote the Mg-Al LDH@Al to release loaded minocycline when the electrode came into contact with the serum fluid. In an in vitro study, almost 100% wipeout of *Escherichia coli* (*E. coli*) and *Staphylococcus aureus* (*S. aureus*) was found 24 h after application of this surface-engineered TENG patch. Elevated fibroblast proliferation and migration was also observed. In the in vivo component of that study, full-thickness skin wounds infected with *S. aureus* were created on the backs of mice. An AC voltage of 0.5–4.5 V and a current of 5–40 nA were collected from the mouse motions via the surface-engineered TENG device. The antibacterial efficacy (96.7% inhibition) and rapid wound healing process both yielded exceptional results compared to the untreated control group (Figure 8(d)). This further realization of medication-containing surface-engineered TENG devices has established a new application route in the field of biomedicine.

## 8. Discussion and conclusions

The impact of delayed wound healing may not be immediately apparent, but a number of complications may arise beyond a higher psychological burden and financial cost. For example, higher morbidity, one small wound leading to devastating consequence as systemic infection or even amputation is not unusual, especially in poorly controlled diabetic or immunocompromised patients. Even in a perfectly healthy young adult, a large wound with or without skin defects after trauma or surgery is medically challenging. Thus, various wound dressings and applications such as negative-pressure devices, hyperbaric oxygen therapy, and electrical stimulation, have been developed prosperously in recent decades.

The therapeutic effects of electrical stimulation have been widely accepted and suggested to accelerate wound healing by some international clinical guidelines [102,103]. However, in clinical practice, electrical stimulation is commonly applicated in rehabilitation, physical therapy, and pain management instead of promoting wound healing. Large size of extracorporeal power supplies is the major reason to frustrate the clinical use for wound healing due to its high-cost, inconvenient, unwearable, not real-time and almost unportable nature. In the recent years, nanogenerators are designed to harvest all possible forms of energy from our daily life into electricity. With a decade’s efforts on more efficient energy transfer, the electricity-generation nanogenerators with biocompatible properties could be integrated into self-powered bioelectrical system for medical applications and wearable self-powered therapeutics. Low cost, portable size and real-time effect are compelling features of nanogenerators-based medical systems to become a promising biotechnology and exert significant impact on wound healing treatment. PENG and TENG, especially, are excellent candidates for self-assisted wound healing systems based on their lightweight, flexibility and elasticity to match with the mechanical motion of human beings [104–106]. Mechanical energy of daily activity and post-traumatic or postoperative rehabilitation could provide as power sources transforming into electrical stimulation for wound healing acceleration in PENG and TENG based medical devices. Indeed, the comprehensive studies of self-powered PENG and TENG have inspired researchers to explore novel ways in the application of biomedicine and self-sustainable therapies. This review summarized the most recent in vitro and in vivo experiments concerning the effectiveness and bio-feasibility of PENG and TENG applications for wound healing. The compatible results of enhanced cellular proliferation, migration and gene expression, shortened wound closure time, and even extended use in the case of infection indicate the advantages of piezoelectric and triboelectric nanogenerators for the wound healing process. Two major effects promoting wound healing through electrical stimulation of PENGs and TENGs are enhanced cellular proliferation, migration [99,107] and anti-bacteria activity [108]. Although the research is in its early stages and results are almost promising, there are still some bottlenecks need to be addressed before actual clinical use. To begin with, the design of nanogenerators should be miniaturized and even customized to match the size of wound and nanogenerators-based wound dressing should attach the wound firmly to keep electrical stimulation with the ability to prevent the influence of body fluid. Then, biocompatibility, non-toxicity, flexibility, elasticity and durability are all necessary for practically adapting PENGs and TENGs into mechanical movement to produce electrical stimulation in the field of wound healing [109,110]. Furthermore, wound exudate and body fluid would corrode the working mechanism of PENGs and TENGs. How to keep energy conversion efficiency and long-time stability under the influence of body fluid is the key challenge of packaging and encapsulation in the in vivo environment. Last but not least, wound healing is a complex and dynamic process. Different wound size and depth need variable strength of electrical stimulation for promoting wound healing [111]. It is difficult to build up a recommended protocol of electrical stimulation for wound healing as researches adopted varying parameters and duration of exposure. Energy output control circuits and the energy conversion efficiency of wearable PENGs and TENGs would also influence wound healing process.

In general, PENGs and TENGs have a promising potential for advanced application in the field of wound healing with many advantages, such as low cost, anti-bacteria, light weight, flexibility and high efficiency. Utilizing the electricity converted from the mechanical energy, PENGs and TENGs could not only supply the power source for medical treatment but also for clinical bio-sensing, which might help us to monitor the change of wound healing. Through this review, we do believe there is still a plenty room for the improvement of nanogenerator application in the field of self-assisted wound healing. Further advanced research and development in this field should be more precise with clinical situations. The technology of PENGs and TENGs would be a revolution in wound management with more efforts on meeting medical needs and commercialization.

## Data Availability

The datasets generated and/or analyzed during the current study are available from the corresponding author on reasonable request.
